# Bacterial outer membrane vesicles bound to bacteriophages modulate neutrophil responses to bacterial infection

**DOI:** 10.3389/fcimb.2023.1250339

**Published:** 2023-10-26

**Authors:** Nina Pennetzdorfer, Medeea C. Popescu, Naomi L. Haddock, Fannie Dupuy, Gernot Kaber, Aviv Hargil, Patrik K. Johansson, Annika Enejder, Paul L. Bollyky

**Affiliations:** ^1^ Division of Infectious Diseases, Department of Medicine, Stanford University, Stanford, CA, United States; ^2^ Immunology Program, Stanford University, Stanford, CA, United States; ^3^ Ecole Normale Supérieure, Paris Sciences et Lettres (PSL) University, Paris, France; ^4^ Geballe Laboratory for Advanced Materials, Stanford University, Stanford, CA, United States; ^5^ Department of Material Science and Engineering, Stanford University, Stanford, CA, United States

**Keywords:** bacteriophages, outer membrane vesicles, *Pseudomonas aeruginosa*, neutrophil influx, innate immunity, host-pathogen interaction, lung infection, cystic fibrosis

## Abstract

*Pseudomonas aeruginosa* is a major human pathogen, particularly effective at colonizing the airways of patients with cystic fibrosis. Bacteriophages are highly abundant at infection sites, but their impact on mammalian immunity remains unclear. We previously showed that Pf4, a temperate filamentous bacteriophage produced by *P. aeruginosa*, modifies the innate immune response to *P. aeruginosa* infections via TLR3 signaling, but the underlying mechanisms remained unclear. Notably, Pf4 is a single-stranded DNA and lysogenic phage, and its production does not typically result in lysis of its bacterial host. We identified previously that internalization of Pf4 by human or murine immune cells triggers maladaptive viral pattern recognition receptors and resulted in bacterial persistence based on the presence of phage RNA. We report now that Pf4 phage dampens inflammatory responses to bacterial endotoxin and that this is mediated in part via bacterial vesicles attached to phage particles. Outer membrane vesicles (OMVs) are produced by Gram-negative bacteria and play a key role in host pathogen interaction. Recently, evidence has emerged that OMVs differentially package small RNAs. In this study, we show that Pf4 are decorated with OMVs that remain affixed to Pf4 despite of purification steps. These phages are endocytosed by human cells and delivered to endosomal vesicles. We demonstrate that short RNAs within the OMVs form hairpin structures that trigger TLR3-dependent type I interferon production and antagonize production of antibacterial cytokines and chemokines. In particular, Pf4 phages inhibit CXCL5, preventing efficient neutrophil chemotaxis in response to endotoxin. Moreover, blocking IFNAR or TLR3 signaling abrogates the effect of Pf4 bound to OMVs on macrophage activation. In a murine acute pneumonia model, mice treated with Pf4 associated with OMVs show significantly less neutrophil infiltration in BAL fluid than mice treated with purified Pf4. These changes in macrophage phenotype are functionally relevant: conditioned media from cells exposed to Pf4 decorated with OMVs are significantly less effective at inducing neutrophil migration *in vitro* and *in vivo*. These results suggest that Pf4 phages alter innate immunity to bacterial endotoxin and OMVs, potentially dampening inflammation at sites of bacterial colonization or infection.

## Graphical Abstract

Graphical abstract—a model for OMV/Pf4 complex-mediated modification of the myeloid cell response to bacterial stimulation. While extruding from its host P. aeruginosa, Pf4 gets associated with OMVs. OMV/Pf4 complexes are endocytosed by lung-resident myeloid cells such as macrophages. Degradation-protected hairpin-structured RNA carried within OMVs triggers TLR3 activation. TLR3 signaling induces type I IFN production which in turn inhibits pro-inflammatory cytokine production in response to other bacterial products. Among the cytokines inhibited are neutrophil chemoattractants such as CXCL1 and CXCL5 resulting in abrogated neutrophil influx to the site of P. aeruginosa infection and eventually to the persistence of the bacteria.

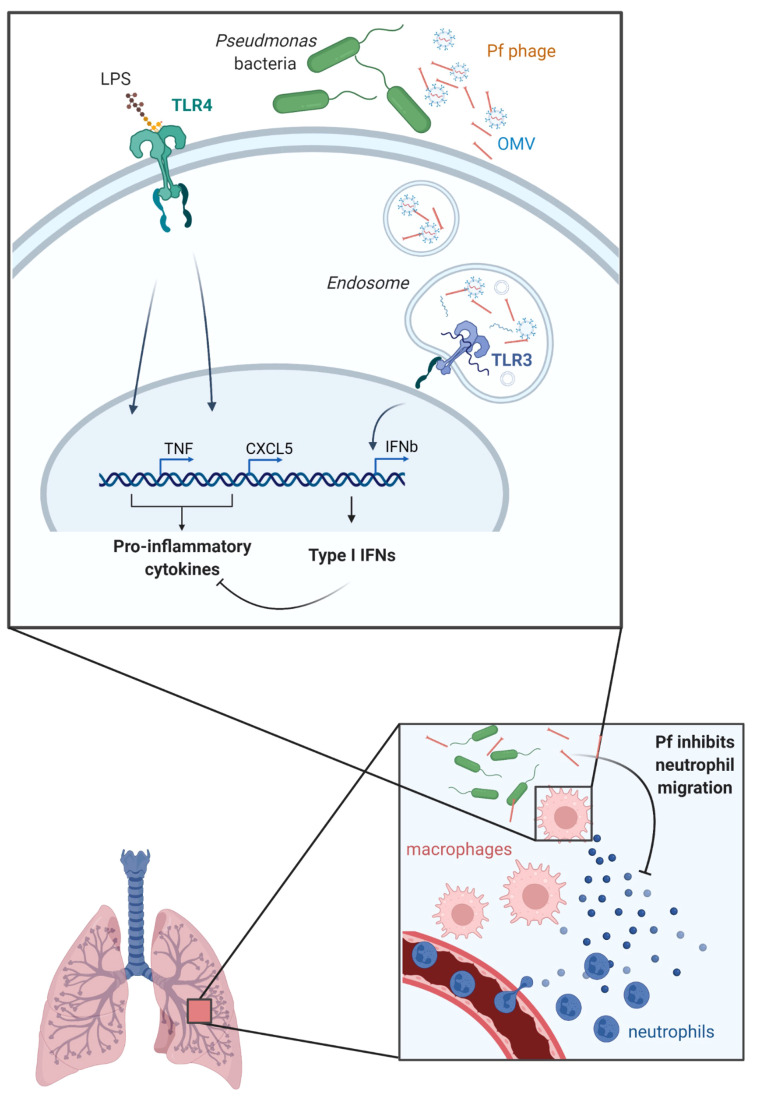

## 1 Introduction

*Pseudomonas aeruginosa* is a versatile and opportunistic pathogen that can cause severe infections in humans, especially in immunocompromised patients. This bacterium has evolved several mechanisms to evade host immune responses, including the formation of biofilms, modifications of lipopolysaccharides (LPS), production of virulence factors, antibiotic tolerance, and secretion of exopolysaccharides. These mechanisms result in persistent bacterial infections, and their understanding is critical for development of effective therapies against *P. aeruginosa* infections.

Bacteriophages, viruses that parasitize bacteria, are abundant at sites of bacterial colonization and infection, including in the lungs of patients infected with *P. aeruginosa*. However, it remains unclear how the innate immune system senses phages or how phages impact sensing of bacteria in the human host (Popescu et al., 2021). While some phages are potent immunogens and have been used for vaccine development (Dąbrowska et al., 2014; De Vries et al., 2021), other phages induce minimal inflammation, including phages used in phage therapy (Cano et al., 2021; Liu et al., 2021). The mechanisms that drive these distinctions are unclear.

The physical association between phages and bacterial debris is an important variable that impacts how the immune system senses phages. Despite being viruses, phages are typically covered in LPS and other poorly characterized bacterial material as they emerge from their bacterial hosts (Van Belleghem et al., 2017). Phages used in phage therapy undergo extensive purification to remove LPS and other impurities (Hietala et al., 2019), whereas this is often not the case with phages used as immunogens (Fogelman et al., 2000). Endogenous phages, produced by our bacterial microbiome, are of course present in their natural state and appear to be well-tolerated in circulation and in our tissues (Popescu et al., 2021).

We and others previously investigated the impact of Pf phages in their bacterial and human hosts. Pf is a genus of filamentous bacteriophages that infect *P. aeruginosa* and includes the phages Pf1 to Pf8 (Knezevic et al., 2015; Mai-Prochnow et al., 2015; Hay and Lithgow, 2019; Fiedoruk et al., 2020). Pf phages are inoviruses and have a single-stranded (ssDNA) genome packaged within a helical filamentous structure made up of multiple subunits of the major coat protein CoaB (Marvin et al., 2014; Hay and Lithgow, 2019; Roux et al., 2019). Pf virions are ∼6–7 nm in diameter and ∼1–2 µm in length (Janmey et al., 2014). Approximately half of *P. aeruginosa* isolates harbor Pf phages (Knezevic et al., 2015; Burgener et al., 2019). Unlike lytic or lysogenic phages that lyse their bacterial hosts after replication, Pf phages follow a chronic infection life cycle whereby Pf virions are continuously extruded from the bacterial cell envelope without lysis (Secor et al., 2020).

Production of Pf4, a well-studied member of the Pf family, contributes to bacterial phenotypes associated with *P. aeruginosa* chronic infection, including reduced twitching motility, increased adhesion, enhanced biofilm formation, and antibiotic tolerance (Rice et al., 2009; Secor et al., 2015; Secor et al., 2017; Sweere et al., 2019; Tarafder et al., 2020). Indeed, filamentous phages like Pf4 contribute to the fitness of their bacterial hosts and the pathogenesis of *P. aeruginosa* infections (Mai-Prochnow et al., 2015; Bille et al., 2017; Pant et al., 2020; Pourtois et al., 2021; Schmidt et al., 2022). Consistent with this, we previously reported that Pf phages are common in individuals with cystic fibrosis and that *P. aeruginosa* infections associated with Pf+ strains are characterized by older aga, advanced lung disease, worse disease exacerbations, and antibiotic resistance in this group (Burgener et al., 2019). Pf phages are likewise associated with chronic *P. aeruginosa* wound infections (Sweere et al., 2019).

Along with effects on bacterial pathogenesis, Pf4 enables *P. aeruginosa* to evade the mammalian host immune response. In a model, of acute lung infection, mice inoculated with *P. aeruginosa* supplemented with Pf4 phage did not develop sepsis, had less pulmonary inflammation, and survived significantly longer than mice infected with WT *P. aeruginosa* only (Secor et al., 2017). In a chronic wound infection model, Pf4 phage was associated with impaired phagocytosis and reduced levels of tumor necrosis factor alpha (TNF-α). These effects were mediated by Toll-like receptor 3 and type I interferon (IFN) production (Sweere et al., 2019). Roles for TLR9, RIG-I, and cGAS/Sting were not observed in that work. However, the nature of these responses was unclear given that Pf4 phage is a ssDNA virus and TLR3 recognizes double-stranded RNAs (dsRNA).

In this study, we investigated the role of Pf4 phage in inhibiting neutrophil chemotaxis in response to bacterial LPS and of bacterial outer membrane vesicles (OMVs) attached to Pf4 phages in these effects. OMVs are small, spherical proteoliposomes (200–400 nm diameter) that are shed from the outer membrane of Gram-negative bacteria, particularly at time of stress or bacterial lysis by phages, including in *P. aeruginosa*. OMVs can contain a range of virulence factors, including LPS, proteins, and nucleic acids. OMVs can activate innate immune cells such as macrophages, dendritic cells, and neutrophils, through the recognition of pathogen-associated molecular patterns (PAMPs) on their surface. LPS molecules present on the surface of OMVs can activate TLR4 on host immune cells, triggering pro-inflammatory cytokine production, e.g., TNF-α or the neutrophil chemoattractant CXCL5 that facilitate bacterial clearance (Schwechheimer and Kuehn, 2015; Roier et al., 2016; Toyofuku et al., 2017).

Our findings provide new insights into the interactions between filamentous phages, OMVs, and host innate immune cells which have important implications for the development of novel therapeutic strategies against *P. aeruginosa* infections.

## 2 Material and methods

### 2.1 Bacterial strains and growth conditions

The *P. aeruginosa* isolate mPAO1 served as a wild type that harbors the filamentous phage Pf4 in all experiments (Holloway et al., 1979). Isogenic phage-free strain PAO1ΔPf4 was derived from PAO1 that lacks Pf4 entirely but can be reinfected by Pf4 (Schmidt et al., 2022). Unless stated otherwise, all *P aeruginosa* strains were grown with aeration in Brain Heart Infusion (BHI, BD Bacto™, Cat. No. 237200), or Luria-Bertani (LB Miller Broth, BD Bacto™, Cat. No. DF0446-07-5) at 37°C.

### 2.2 Preparation and quantification of bacteriophages

*P. aeruginosa* mPAO1 was used to produce Pf4. Mock phage preparations from PAO1ΔPf4 were prepared according to the same protocol as other phage samples. Cultures were inoculated with an OD_600_ of 0.1 and grown to the mid-log phase in which the respective phage was added and co-incubated overnight. The next day, the bacteria were harvested at 6,000 × *g*; 30 min; 4°C. The supernatant was sterile filtered using a 0.22-µm bottle top filter and treated with 50 U/ml of benzonase nuclease (Sigma-Aldrich, Cat. No. E1014-25KU) or RNase-free DNase I nuclease (Qiagen, Cat. No. 79254) at 37°C for 2 h. An aliquot of the phage solution was left untreated (impure phage lysate). The remaining phage solution was incubated with polyethylene glycol 8000 (PEG-8000, Sigma-Aldrich, Cat. No. P2139-500G) at 4°C overnight either once (1× PEG) or twice (2× PEG) as described previously (Boulanger, 2009) and dialyzed against PBS for 48 h. Purified phage preparations were quantified by plaque assays as well as by qRT-PCR using primers directed against the major coat protein of the respective phage and a vector based (pBS-SK back bone) standard comprising the gene encoding for the respective major coat protein. Phages were stored in SM-buffer or 1× PBS at 4°C. Endotoxin was quantified by EndoZyme II assay (BioVendor, Cat. No. 890030) as per manufacturer’s directions.

#### 2.2.1 Plaque assays

Plaque assays were performed using ΔPf4 as an indicator strain grown on LB plates. Phages in filtered supernatants, 1× PEG or 2× PEG treated, were serially diluted 10× in PBS and spotted onto lawns of the indicator strain. Plaques were counted after 18 h of growth at 37°C.

#### 2.2.2 Pf4 phage virion quantification by qRT-PCR

Pf4 virion copy number was measured using qRT-PCR as previously described (Burgener et al., 2020). Briefly, filtered supernatants, 1× PEG or 2× PEG preparations, were treated with DNase I (10 µl of a 10-mg/ml stock per ml supernatant) at 37°C followed by incubation at 70°C for 10 min to heat inactivate the DNase I. 10-µl reaction volumes contained 5 µl Luna^®^ Universal One-Step Mix (New England Biolabs, Cat. No. E3005L), 100 nM *coaB* primer, and 2 µl supernatant, 1× PEG or 2× PEG Pf4 preparation. Cycling conditions were set to the following: 50°C 2 min; 95°C 2 min; (95°C 15 s; 60°C 1 min) × 40 cycles. A standard curve was constructed using a plasmid containing the template sequence at a known copy number per ml. Pf4 copy numbers were then calculated by fitting Ct values of the unknown samples to the standard curve.

### 2.3 Preparation and quantification of OMVs

Overnight cultures of the respective strains were cultivated in BHI. 3 × 350 ml BHI in 1 l flasks were inoculated with 3.5 ml ONC and were incubated for 16 h with aeration at 37°C until they reached an OD_600_ greater than 4. The cultures were separated by centrifugation at 6,000 × *g* for 60 min at 4°C. The supernatant was sterile filtered using a 0.22-µm bottle top filter to remove any intact cells. To ensure that no bacteria remained in the filtered supernatant, a 1-ml aliquot was plated onto LB agar plates and incubated at 37°C overnight. The OMVs in the supernatant were harvested by ultracentrifugation 150,000 × *g* for 4 h at 4°C as previously described (Schild et al., 2008; Kohl et al., 2018). The OMV pellets were resuspended in 1× PBS and were stored at 4°C. The protein concentration was determined by BCA protein assay (Thermo Scientific, Cat. No. 23227) according to the manufacturer’s protocol. The LPS concentration was quantified by using the Purpald assay as described previously (Lee and Tsai, 1999; Kohl et al., 2018).

### 2.4 Fluorescent labeling of Pf4 and OMVs

2× PEG-treated Pf4 lysate was labeled with Alexa Fluor 488 TFP ester (Molecular Probes, Cat. No. A37570), and OMVs were labeled with either DiD′ (1,1′-dioctadecyl-3,3,3′,3′-tetramethylindodicarbocyanine, 4-chlorobenzenesulfonate salt, Thermo Fisher Scientific, Cat. No. D7757), SYTO RNASelect (Thermo Fisher Scientific, Cat. No. S32703), and BODIPY (Thermo Fisher Scientific, Cat. No. D2184) according to the manufacturer’s instructions. Unincorporated dye was removed by three ultracentrifugation rounds (150,000 × *g*, 1 h, 4°C). Labeled OMVs and Pf4 were stored in 1 × PBS at 4°C.

### 2.5 Confocal microscopy of macrophages

1.5 × 10^4^ RAW 264.7 murine macrophages were seeded onto collagen-coated acid-etched cover slips, primed with 100 ng/ml LPS, and incubated at 37°C and 5% CO_2_ to adhere overnight. Purified Pf4 preparation at a concentration of 10^10^ pfu/ml and/or OMVs at a concentration of 1 μg/ml (by protein) were then co-incubated with RAW 264.7 at 37°C and 5% CO_2_ for 1 h. Subsequently, cells were fixed, permeabilized at room temperature for 20 min, stained with phalloidin-CF555 conjugate, and incubated with 1 µg/ml DAPI at room temperature for 10 min. The cover slip was transferred onto a glass slide using ProLong Gold Mountant (Invitrogen Cat. No. P36930), and confocal microscopy was performed.

### 2.6 Flow cytometry

2.5 × 10^5^ U937 macrophages were seeded per well into a 24-well plate and were either left untreated or treated with 10^11^ pfu/ml AlexaFluor488-labeled Pf4 with or without 1 μg/ml DiD-labeled OMV, or RNASelect-labeled OMV (by protein) at 37°C and 5% CO_2_ for 1.5 h. When uptake inhibitor treatments were used, cells were pretreated with 100 ng/ml wortmannin (Sigma-Aldrich, Cat. No. W1628-1MG), 1 µg/ml nocodazole (Fisher Scientific, Cat. No. AC358240100), 5 µM chlorpromazine (Sigma-Aldrich, Cat. No. C8138-5G), and 2 µM cytochalasin D (Sigma-Aldrich, Cat. No. C8273-1MG). Samples were stained with Zombie Near-IR Live-Dead dye (BioLegend, Cat. No. 423105) and run live on a Cytek Northern Lights 3000 flow cytometer. Data were analyzed on FlowJo™ software.

### 2.7 Tissue culture

Human U937 macrophages (ATCC, Cat. No. CRL-1593.2™) were maintained in RPMI (Thermo Fisher Scientific, Cat. No. 11875093) supplemented with 10% FBS (RMBIO, Cat. No. FGR-BBT), penicillin–streptomycin (Fisher Scientific, Cat. No. MT30002CI), and sodium pyruvate (Thermo Fisher Scientific, Cat. No. 11360070). Murine RAW 264.7 (ATCC, Cat. No. YIB-71™) were maintained in DMEM (Thermo Fisher Scientific, Cat. No. 11-995-065) supplemented with 10% FBS (RMBIO, Cat. No. FGR-BBT), penicillin–streptomycin (Fisher Scientific, Cat. No. MT30002CI), and sodium pyruvate (Thermo Fisher Scientific, Cat. No. 11360070). HEK-Blue hTLR3 (InvivoGen, Cat. No. hkb-tlr3) and HEK-Blue IFNα/β (InvivoGen, Cat. No. hkb-ifnab) were maintained in DMEM as above, with the addition of 30 µg/ml of blasticidin (InvivoGen, Cat. No. ant-bl-05) and 100 µg/ml of Zeocin™ (InvivoGen, Cat. No. ant-zn-05) as per the manufacturer’s instructions.

#### 2.7.1 Cell stimulation

For cytokine release and reporter assays, cells were seeded at a density of 5 × 10^4^ cells/well in a 96-well plate. Unless otherwise specified, stimuli were as follows: 1 × 10^8^ pfu/well of purified phage preparation, 10 ng/ml purified OMVs, 100 ng/ml LPS (InvivoGen, Cat. No. tlrl-eblps), and 10 μg/ml high molecular weight poly (I:C) (InvivoGen, Cat. No. tlrl-pic). The TLR3 signaling inhibitor (EMD Millipore, Cat. No. 614310) was used at 27 μM. Cells were incubated at 37°C and 5% CO_2_ for the indicated timepoints and then centrifuged at 300 × *g* for 5 min prior to supernatant removal.

#### 2.7.2 Enzyme-linked immunosorbent assay

Human CXCL5 (BioLegend; Cat. No. 440904), human TNF-α (BioLegend; Cat. No. 430904), human IFNβ (Invitrogen; Cat. No. 414101), mouse CXCL1 (BioLegend; Cat. No. 447504), mouse CXCL5 (Thermo Fisher Scientific; Cat. No. EMCXCL5), and mouse TNFα (BioLegend; Cat. No. 430904) were performed using the manufacturer’s instructions. Absorbance was read on a Spark microplate reader (Tecan).

#### 2.7.3 Lactate dehydrogenase cytotoxicity assay

Cytotoxicity was determined using the LDH-Glo kit (Promega, Cat. No. J2380) as per the manufacturer’s instructions. Absorbance was read on a Spark microplate reader (Tecan).

#### 2.7.4 Luminex analysis

U937 macrophages were plated in 96-well plates at 5 × 10^4^ cells/well. Cells were stimulated for 24 h with 1 × 10^9^ pfu/well of purified Pf4 phage or equivalent volume ΔPf4 preparation, 10 ng/ml purified OMVs, and 100 ng/ml LPS (InvivoGen, Cat. No. tlrl-eblps). Cells were centrifuged at 300 × *g* for 5 min, and supernatant was collected and used for cytokine profiling through Luminex (The Human Immune Monitoring Center, Stanford). Human 89-plex kits were purchased from eBioscience/Affymetrix and used accordingly to the manufacturer’s recommendations with modifications as described below. Briefly, beads were added to a 96-well plate and washed in a BioTek ELx405 washer. Samples were added to the plate containing the mixed antibody-linked beads and incubated at room temperature for 1 h followed by overnight incubation at 4°C with shaking. Cold- and room-temperature incubation steps were performed on an orbital shaker at 500–600 rpm. Following the overnight incubation, plates were washed in a BioTek ELx405 washer and then biotinylated detection antibody added for 75 min at room temperature with shaking. The plate was washed as described above, and streptavidin-PE was added. After incubation for 30 min at room temperature, washing was performed as above and the reading buffer was added to the wells. Each sample was measured in duplicate. Plates were read using a Luminex 200 instrument with a lower bound of 50 beads per sample per cytokine. Custom assay Control beads by Radix Biosolutions were added to all wells.

##### 2.7.4.1 Luminex data analysis

Technical replicate MFI values were averages for each sample and transformed into log_2_ fold change with respect to the control LPS sample. Data were plotted as a heatmap using the heatmap package (v1.0.12) in R.

### 2.8 Coherent anti-Stokes Raman scattering and confocal microscopy of Pf4

1× PEG Pf4 phages in PBS were adsorbed onto coverslips and characterized *in situ* by coherent anti-Stokes Raman scattering (CARS) microscopy with an inverted microscope (Ti2 Eclipse with a C2 confocal scanning head and a Nikon CFI Apochromat TIRF 100XC Oil objective). The vibrations were coherently driven by two laser beams overlapped in time and space, generated by a ps-pulsed laser system (APE picoEmerald S, 2-ps pulse length, 80-MHz repetition rate, and 10-cm^−1^ bandwidth) with a 1,031-nm Nd:VAN laser and an optical parametric oscillator (OPO), tunable between 700 and 960 nm (pumped with the second-harmonic generation of the 1,031-nm laser). The OPO wavelength was set to 797 nm to address the lipid-specific symmetric stretching vibration of CH_2_ at 2,848 cm^−1^. The quadratic dependence of the CARS signal on the number density of the probed vibrational group provides sharp contrast for the lipid-dense regions without the need for external labels. The excitation powers at the sample were 13 mW for the 1,031-nm beam and 27 mW for the OPO beam, and CARS signals were collected in the forward direction with a photomultiplier tube (Hamamatsu, R6357). Images with multiple phages within the field of view were captured in a single focal plane with 10.8-μs dwell time and 20-nm pixel size. The acquired images were prepared and analyzed in ImageJ. After east shadow correction, the images were bandpass Fourier filtered between 2 and 100 pixels with 5% tolerance of direction and suppression of horizontal stripes. A Gaussian blur filter (sigma = 1.5) was applied, and the background was subtracted by a rolling ball (radius 80 pixels). For spectral acquisitions, Z-stacks (15 slices) were collected on individual phages (n = 4) with 5.3-μs dwell time, 12-nm pixel size, and 0.2-μm vertical separation between adjacent z-positions. The stacks were east shadow corrected, and the total intensity for a region of interest (ROI) manually created around the phage was evaluated for a range of vibrations (with the OPO tuned from 800 to 785.5 nm in steps of 0.5 nm) in the C-H_x_ stretching region. The background signal from water was evaluated in ROIs of similar areas a distance away from the phage. The background had no discernable peaks and was subtracted from the spectrum. On the same microscope platform, equipped with a LU-N4 excitation source and a C2-DUVB GaAsP detector unit, the fluorescence from phages labeled with AF514 were collected with an excitation wavelength of 488 nm and 10.8-µs dwell time and 20-nm pixel size. The confocal fluorescence images were processed in ImageJ with a smoothing function and a rolling ball (radius 80 pixels) background subtraction.

### 2.9 Transmission electron microscopy and immunogold labeling of OprF

5 µl of phage lysates and OMV preparations (~50 µg/ml protein) were absorbed onto glow discharged formvar and carbon-coated copper grids (300 nm mesh size, Electron Microscopy Sciences, Cat. No. FCF300-CU-50) at room temperature for 5 min, negatively stained with 1% (w/v) uranyl acetate at room temperature for 1 min, and imaged with a JEM-1400 transmission electron microscope (JEOL) operated at 120 kV. The images were taken with a Gatan Multiscan 791 CCD camera. Immunogold labeling of OMV was performed using 5 µl of 1× PEG-treated Pf4 lysates (~50 µg/ml protein) that were absorbed onto formvar- and carbon-coated copper grids at room temperature for 5 min and subsequently blocked in 2.5% goat serum (Fisher Scientific, Cat. No. NC9678224) at room temperature for 15 min. The samples were incubated in α-OprF rabbit serum (10 µg/ml, MyBioSource, Cat. No. MBS7113710) at room temperature for 15 min, followed by an incubation in goat-anti-rabbit IgG serum conjugated to 10-nm gold particles (1:50, Abcam, Cat. No. ab39601) for 15 min and negative staining in 1% (w/v) uranyl acetate for 1 min.

### 2.10 RNA isolation from OMVs

RNA was extracted from OMV preparations isolated from cultures of PAO1 or PAO1 ΔPf4. Each preparation was split into three aliquots which were incubated at 37°C for 1 h with either RNase free DNase I (QIAGEN, Cat. No. 1080901), proteinase K (Bioline, Cat. No. BIO-37084), and RNase free DNase I or left untreated. OMVs were disrupted by adding 3% (w/v) SDS (Sigma-Aldrich, Cat. No. 75746-1KG). The hot phenol extraction protocol initially published by Habier et al. (2018) was modified using TRI Reagent® (Sigma-Aldrich, Cat. No. T9424). After chloroform purification and ethanol precipitation, the extracts were further processed and purified using RNeasy columns (RNeasy Mini Kit, QIAGEN, Cat. No. 74106) and washed using buffers RW1 and RPE (RNeasy Mini Kit, QIAGEN, Cat. No. 74106) as per the manufacturer’s instructions and eluted in RNase-free water. 100 ng/ml RNA of the respective extractions was used in tissue culture experiments.

### 2.11 OMV RNA sequencing and analysis

After RNA extraction from OMVs, the samples were depleted from rRNA using the RiboMinus™ Bacteria 2.0 Transcriptome Isolation Kit (Thermo Scientific; Cat. No. A47335). The samples were further enriched for small RNA species (<200 nt) using the RNA Clean and Concentrator Kit (Zymo Research; Cat. No. R1013). Sequencing libraries were prepared with the KAPA Total RNA Kit for Stranded RNA HyperPrep (Roche; Cat. No. 07962142001) and sequenced by on the Illumina platform (Sjöström et al., 2015).

Quality control was performed on raw data using FASTQC 0.11.9 and MULTIQC (Ewels et al., 2016). Adapter sequences and low-quality reads were trimmed using Trimmomatic 0.39 (Bolger et al., 2014) with settings 2:30:10:2:keepBothReads LEADING:3 TRAILING:3 SLIDINGWINDOW:4:15 MINLEN:36. Quality control was performed again on trimmed reads, and overrepresented sequences were extracted from FASTQC report output. Overrepresented sequences had secondary structures predicted using Vienna RNAfold (Lorenz et al., 2011) with default settings. Free energies were obtained from RNAfold outputs and plotted in GraphPad Prism, and a Gaussian nonlinear regression applied to generate a fitted distribution line for PAO1 and PAO1ΔPf4 free energy distributions.

### 2.12 Preparation of whole-cell lysates, SDS-PAGE, and immunoblot

For whole-cell lysate (WCL) preparation, equal amounts of cells (equivalent to 1.5 ml of a culture with an OD_600_ of 1) were pelleted by centrifugation (6,000 × *g*, 5 min, 4°C) from the respective *P. aeruginosa* culture. Cell pellets were resuspended in 60 µl of 5× sample buffer according to Laemmli, boiled for 10 min at 100°C, and either used for SDS-PAGE or stored at −20°C (Laemmli, 1970).

Proteins contained in WCL or OMVs were separated by sodium dodecyl sulfate-polyacrylamide gel electrophoresis (SDS-PAGE) using SurePAGE™, Bis-Tris (4%–12%, GenScript, Cat. No. M00654), and MES SDS running buffer (GenScript. Cat. No. M00677). The Chameleon^®^ Duo Pre-stained Protein Ladder (8 to 260 kDa, LI-COR Biosciences, Cat. No. NC0738562) was used as a molecular mass standard. Subsequently, protein bands were stained with Coomassie solution (Kang et al., 2002) or further processed for immunoblot analysis. Protein bands were transferred to a LI-COR Odyssey Nitrocellulose membrane (LI-COR Odyssey, Cat. No. 926-31092). After blocking in a casein-BSA buffer at 4°C overnight, the membranes were incubated with α-OprF rabbit serum (MyBioSource, Cat. No. MBS7113710) diluted 1:2,500 in casein-BSA buffer for 2 h at room temperature. After several washing steps in TBS-T and TBS, the membranes were incubated with the secondary antibody donkey anti-rabbit IgG conjugated to IRDye^®^ 680RD (Li-COR Biosciences, Cat. No. 926-68073) 1:20,000 diluted in casein-BSA buffer for 30 min at room temperature. Reactive protein bands were detected by using the LI-COR Odyssey 9120 imaging system.

### 2.13 Isolation and preparation of neutrophils

Human neutrophil isolation protocol was adapted from Swamydas et al. (Swamydas et al., 2015). Briefly, whole blood was obtained from the Stanford Blood Center, diluted 1:1 in 1× PBS (Corning, Cat. No. 21-040-CV), and layered over a double Histopaque-based density gradient density 1.119 g/ml (Sigma-Aldrich, Cat. No. 11191-6X100ML) and density 1.077 g/ml (Sigma-Aldrich, Cat. No. 10771-6X100ML). The suspension was centrifuged at 900 *x g* for 30 min at 22°C with no brake, and neutrophils from the interface of the Histopaque-1119 and Histopaque-1077 layers were collected and washed in RPMI + 10% FBS. Count and viability of neutrophils was determined by trypan blue exclusion.

### 2.14 Murine pneumonia model

Experiments were performed as described in Secor et al.(Secor et al., 2017). Briefly, for the live bacterial inoculum, an isolated colony of *P. aeruginosa* PAO1 or PAO1ΔPf4 was grown to the mid-exponential phase (OD_600_, 0.5) in 4 ml LB broth at 37°C with aeration. The PAO1 culture was infected with 100 µl of Pf4 stock (10^10^ pfu/ml) or PBS control to promote phage production. These cultures were grown overnight at 37°C with aeration. On the following day, bacteria were pelleted by centrifugation at 6,000 × *g* for 10 min, washed three times in sterile PBS, and resuspended in PBS to a final concentration of 3 × 10^8^ CFU/ml. Mice (8–10 weeks C57BL/6, male; The Jackson Laboratory) were intranasally inoculated with 1.5 × 10^7^ CFU/ml in 50 µl PBS of each strain or PBS control.

For the inocula using isolated phages and/or OMVs, either 10^9^ pfu/ml of 1× PEG or 2× PEG Pf4, 10 ng/ml OMVs, or 10 ng/ml LPS were prepared in sterile PBS. Mice were intranasally inoculated with 50 µl of the respective inoculum.

Mice were sacrificed at 24 h post infection. The lungs were lavaged with 0.8 ml sterile PBS as described previously (Secor et al., 2017). BAL fluid samples were pelleted, and supernatant was frozen for use in enzyme-linked immunosorbent assay (ELISA) and qPCR assays. BAL cells were resuspended in 1 ml ACK lysis buffer for 30 s, followed by washing in PBS with 2% fetal calf serum and 1mM EDTA (fluorescence-activated cell sorting [FACS] buffer). The cells were resuspended in 1 ml FACS buffer and stained with Zombie Near-IR Live/Dead (BioLegend, Cat. No. 423105), CD11b-Pacific Blue (BioLegend, Cat. No. 101223), CD11c-BV605 (BioLegend, Cat. No. 117333, CD45.2-BV650 (BioLegend, Cat. No. 109835), Ly6G-BV785 (BioLegend, Cat. No. 127645), Siglec F-PE (BioLegend, Cat. No. 155505), and Ly6C-AF647 (BioLegend, Cat. No. 128009) as per manufacturer’s instructions. After staining, the cells were fixed with 4% paraformaldehyde and then run on a Cytek Northern Lights machine, followed by analysis with FlowJo™ software. Total live cells within the CD45^+^ gate was reported, as well as live neutrophils within the CD45^+^ Ly6G^+^ gate. Absolute cell counts were adjusted by total BAL volume recovered.

### 2.15 Statistical analysis

Where n is not stated, graphs show a representative experiment of n ≥ 3 assays, with n ≥ 3 technical or biological replicates. All statistical analyses were performed using GraphPad Prism (GraphPad Software, Inc. La Jolla, CA). Significance was assessed by ANOVA followed by Holm–Šídák test for multiple comparisons unless otherwise indicated. Depicted are means with standard deviation (SD) of the replicates unless otherwise stated. Statistical significance was considered *p* < 0.05. Non-significance was indicated by the letters “n.s.”. For experiments involving cell assays, each replicate was normalized against a specified control condition to control for varying inter-experiment intensities of cytokine production or reporter protein secretion.

## 3 Results

### 3.1 Pf4 phages are associated with *P. aeruginosa* OMVs

Since Pf phages are ssDNA viruses and TLR3 is a sensor for dsRNA, it was unclear how these responses are mediated (Sweere et al., 2019). Given that phages of Gram-negative bacterial hosts are covered in LPS and the necessity to undergo extensive purification to remove these impurities before being able to administer these phages as therapies in patients (Van Belleghem et al., 2017; Hietala et al., 2019), we examined whether phages are associated with any other bacterial structures.

**Figure 1:**
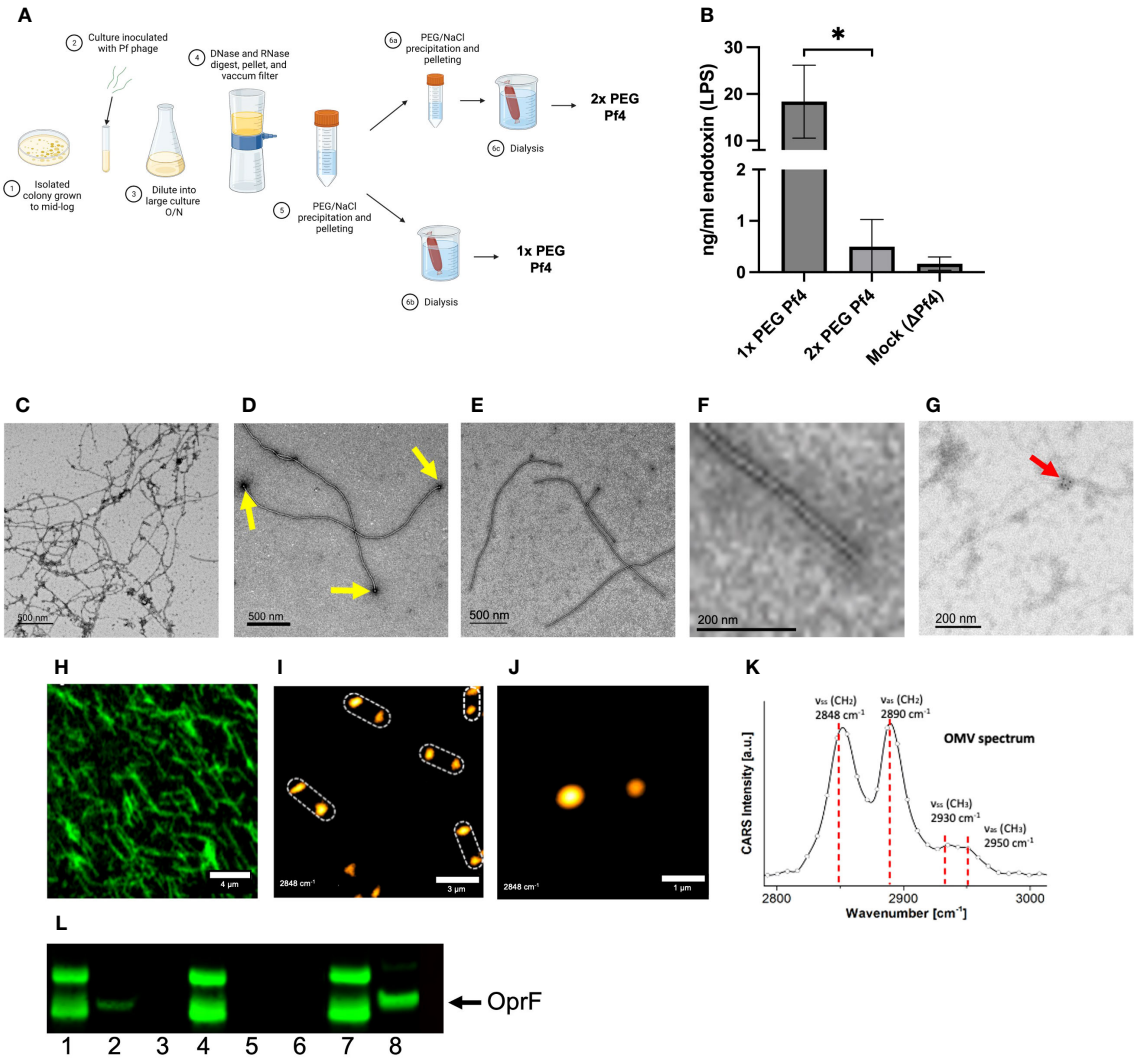
Pf4 phages are associated with *P. aeruginosa* outer membrane vesicles (OMVs). **(A)** Schematic of Pf4 phage production procedure of 1× PEG and 2× PEG phages. The supernatants of large-scale superinfected PAO1 cultures are sterile filtered, digested with DNase I or the non-specific endonuclease benzonase, and subsequently pelleted by centrifugation. The Pf4 phage lysate is precipitated with polyethylene glycol (PEG) and dialyzed, resulting in 1× PEG Pf4. For 2× PEG Pf4 preparation, the PEG precipitation is repeated a second time. **(B)** Endotoxin concentrations for various Pf4 preparations at the relevant dilutions used in experiments. **(C–F)** Transmission electron micrographs derived from *P. aeruginosa* PAO1 of **(C)** crude lysate with Pf4 phage associated with OMVs, **(D)** 1× PEG-treated Pf4 associated with OMVs (indicated by yellow arrows), **(E)** 2x PEG Pf4 sample displayed at lower, and **(F)** higher magnification. **(G)** Transmission electron micrograph of 1× PEG Pf4 with OMVs stained with anti-OprF antibody followed by gold-labeled anti-rabbit IgG (indicated by the red arrow). **(H)** Confocal microscopy of Alexa Fluor 514-labeled 1× PEG Pf4. The same sample was used to perform coherent anti-Stokes Raman scattering (CARS) microscopy at a lower **(I)** and higher resolution **(J)**, indicating the presence of colocalized lipid assemblies with diameters of 0.4–0.7 µm at 1.5–2.5 µm, the approximate length of the filamentous phage Pf4. **(K)** Pf4 phages analyzed with spectral CARS microscopy reveal peaks at 2,848 cm^−1^, 2,890 cm^−1^, 2,930 cm^−1^, and 2,950 cm^−1^. These are typical signatures for lipids and lipopolysaccharide (LPS) of which OMVs are composed of. **(L)** Immunoblot analyses processed with anti-OprF antiserum of (1) PAO1 whole-cell lysate (WCL), (2) 1× PEG Pf4, (3) 2× PEG Pf4, (4) ΔPf4 WCL, (5) 1× PEG ΔPf4, (6) 2× PEG ΔPf4, (7) PAO1 WCL, and (8) OMVs derived from PAO1. All data are shown as mean ± SD of three biological replicates and three technical replicates per condition and experiment. *p < 0.05. Significance was assessed by ANOVA followed by the Kruskal–Wallis test.

To test this, we evaluated the impact of performing one versus two rounds of polyethylene glycol (PEG) precipitation on our phage preparations (**Figure 1A**). The endotoxin content of a 1× PEG-treated phage preparation was significantly higher than the Pf4 preparation that underwent a second round of PEG precipitation. We also describe “mock preparations” collected from PAO1 ΔPf4 supernatants as controls which were processed in the same manner than Pf4 preparations (**Figure 1B**).

Transmission electron microscopy (TEM) was used to examine isolated Pf4 phages. Spherical structures were observed on the distal ends of Pf4 (**Figures 1C, D**). We found that 2× PEG Pf4 were devoid of these structures as assessed by TEM (**Figures 1E, F**). Bacterial OMVs are known carriers of LPS, nucleic acids, and protein cargo. OMVs bud off of the bacterial outer membrane at times of stress (Koeppen et al., 2016; Roier et al., 2016), including during phage production. Both DNA and RNA are reported to be bound to the surface of OMVs as well as contained inside (Ghosal et al., 2015; Han et al., 2019; Lee, 2019; Augustyniak et al., 2022). We therefore further examined whether OMVs are associated with Pf4 phages and confirmed the identity of OMVs of 1× PEG Pf4 by immunogold labeling. OprF is an outer membrane porin of *P. aeruginosa* which tethers the outer membrane to the peptidoglycan layer and regulates OMV production (Augustyniak et al., 2022). The presence of OprF was observed by using an anti-OprF antibody and an immunogold-labeled secondary antibody for TEM imaging. OprF was found to appear in clusters at the end of Pf4 phage particles (**Figure 1G**) and was also confirmed by immunoblot analysis using anti-OprF serum (**Figure 1L**).

A second orthogonal imaging approach, coherent anti-Stokes Raman scattering (CARS) microscopy for label-free visualization of lipids, was used to confirm the presence of lipid deposits at the end of Pf4 particles. CARS is a microscopy tool for label-free visualization of lipid bilayers that could be expected to reveal the presence of OMVs. CARS imaging of 1× PEG Pf4 indeed indicated the presence of large multilayer lipid droplets on the distal ends of the filamentous phage particles (**Figures 1H–K**). These lipid droplets were separated by approximately 2 µm along a linear axis—the same length as a Pf4 phage particle.

### 3.2 Pf4 alters internalization patters and intracellular trafficking of OMVs in macrophages

Other groups have reported that OMVs produced by *P. aeruginosa* strain PAO1 are taken up by mammalian cells via lipid rafts and membrane fusion, leading to cytosolic localization (Bomberger et al., 2009; O’Donoghue and Krachler, 2016). In contrast, we previously showed that Pf4 phage is endocytosed and traffics through endosomal vesicles in mammalian cells (Sweere et al., 2019). This raised the possibility that Pf4 phage altered the manner of internalization of OMVs. Endosomally internalized OMVs would be more likely to engage endosome restricted TLR3.

**Figure 2:**
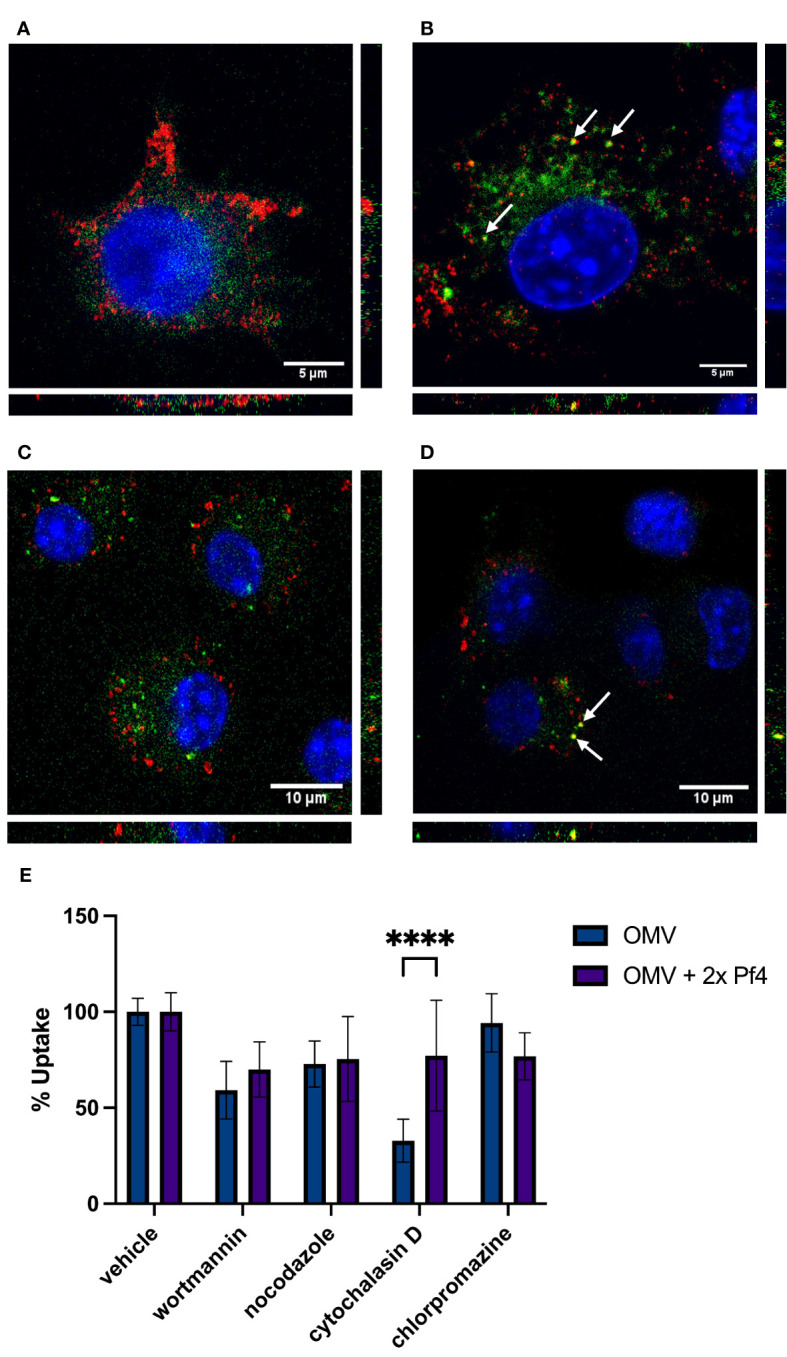
Pf4 phage alters patterns of OMV internalization and trafficking. **(A, B)** Mouse RAW macrophages treated with DiD-labeled OMV (green) **(A)** and DiD-OMV (green) + unlabeled 2× PEG Pf4 **(B)**. Cells were stained with anti-TLR3 antibody (red) and DAPI (blue). **(C, D)** Mouse bone-marrow-derived macrophages treated with BODIPY-labeled OMV (green) **(C)** and BODIPY-OMV (green) + unlabeled 2× PEG Pf4 **(D)**. Cells were stained with anti-EEA-1 antibody (red) and DAPI (blue). **(E)** OMV, or 2× PEG Pf4 + OMV uptake by human U937 macrophages in absence or presence of the indicated uptake inhibitors were determined by flow cytometry. All data are shown as mean ± SD of at least three biological replicates with n ≥ 3 technical replicates per condition and experiment. Statistical significance for the data in this panel was determined using a paired T-test. ****p < 0.0001.

We visualized macrophages exposed to labeled OMVs as well as to OMVs complexed with unlabeled 2× PEG Pf4 and found that the internalization of OMVs differ based on association with Pf4 (**Figures 2A–D**). OMVs alone appear to accumulate diffusely in the cytosol, whereas Pf4-OMV complexes traffic analogously to our previously reported findings on Pf4 phage and enter endosomes, displayed by the co-localization with TLR3 (**Figure 2B**), and the early endosomal marker EEA-1 (**Figure 2D**).

As an additional confirmation of altered OMV uptake in the presence of Pf4, we performed flow cytometry to determine what inhibitors are capable of blocking OMV uptake by macrophages (**Figure 2E**). We use four inhibitors each having different specificities for uptake pathways. Wortmannin, an inhibitor of the phosphatidyl inositol 3 kinase pathway, blocks clathrin-independent endocytosis. Nocodazole inhibits microtubule polymerization and therefore blocks endocytosis of large particles. Cytochaliasin D inhibits actin polymerization and has been shown to block lipid raft-mediated membrane fusion uptake. Finally, chlorpromazine inhibits clathrin-mediated endocytosis. We found that OMVs alone are most efficiently blocked from entering cells by the lipid raft inhibitor cytochalasin D (**Figure 2E**). When 2× PEG Pf4 phage is present, this inhibitor is much less effective at blocking OMV uptake, indicating a shift toward endosomal uptake. Indeed, wortmannin is the most effective inhibitor of OMV/Pf4 complexes (**Figure 2E**).

Together, these data demonstrate that binding to Pf4 phage alters the uptake pattern of OMVs, allowing trafficking of vesicles where TLR3 is present. In this way, Pf4 phages alter the innate immune perception of bacterial products.

### 3.3 Pf4-associated OMVs contain small RNAs which induce TLR3 and type I interferon

**Figure 3:**
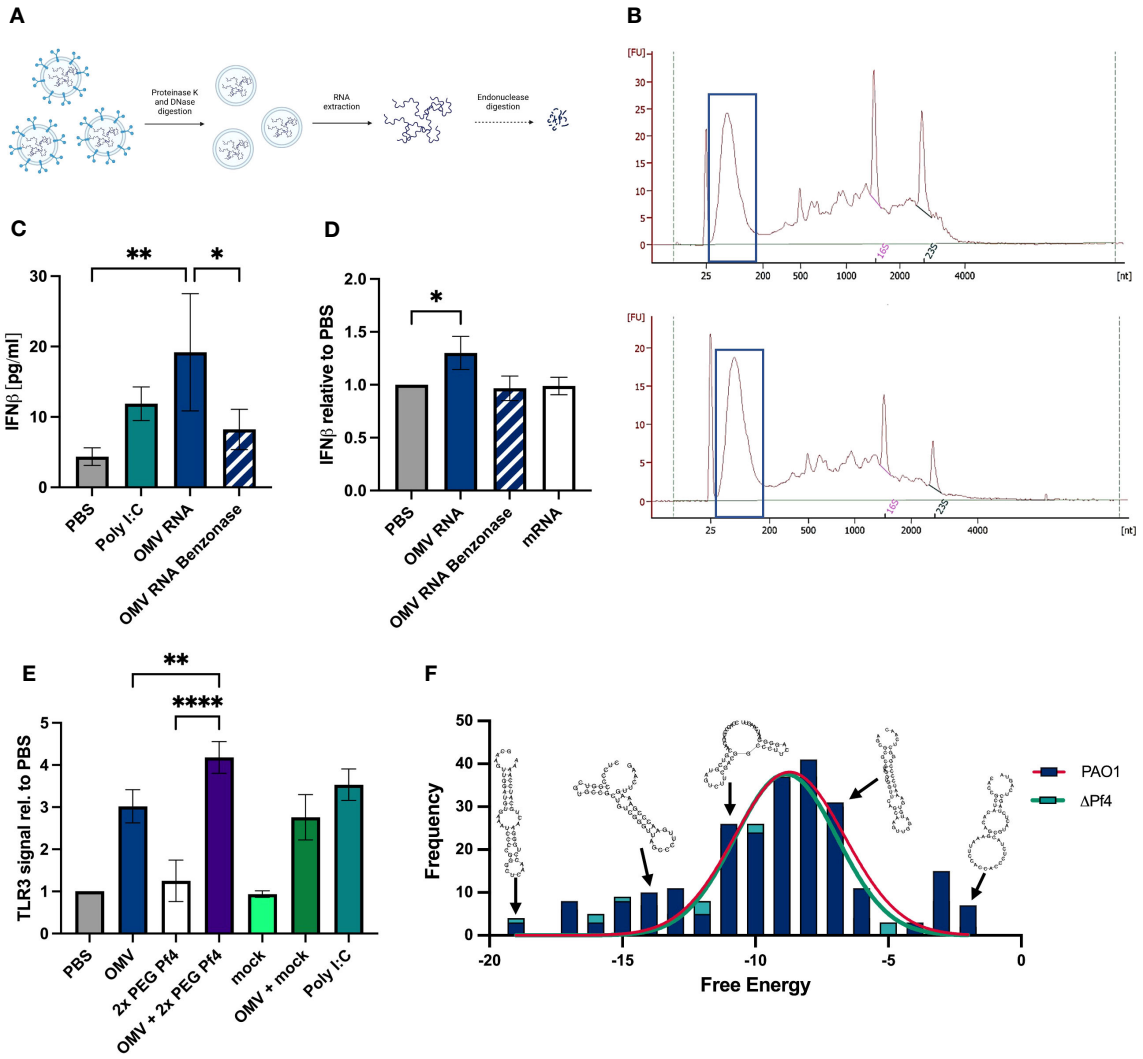
OMVs contain small RNAs that induce type I IFN production. **(A)** Schematic representation of the sRNA isolation from OMVs: OMVs derived from ΔPf4 were isolated, treated with either proteinase K or DNase I prior to RNA extraction with TRIzol®, rRNA depletion, and enrichment of small RNAs (for details please see Material and Methods). **(B)** Representative bioanalyzer trace of ΔPf4 OMVs (top) and mPAO1 OMVs (bottom) RNA preparations with small RNA fractions framed. **(C)** Human U937 macrophages were stimulation with 100 ng/ml of purified RNA derived from OMVs for 24 h, and secreted IFNβ was quantified by ELISA. As a negative control, OMV-derived RNA was digested with benzonase for 24 h. **(D)** Purified RNA was transfected into human U937 macrophages using RNAfectamine, a cationic lipid transfection agent. Cells were incubated for 24 h, and supernatants were recovered. HEK-IFNα/β cells were incubated for 24 h with supernatants, and IFN production was determined by absorbance and normalized to the PBS control condition. **(E)** Conditioned media from human U937 macrophages stimulated for 24 h with the indicated substances were added to HEK-Blue TLR3 reporter cells. TLR3 activation relative to PBS is displayed. **(F)** Histogram of free energies of predicted secondary structures for transcripts comprising >0.1% of reads in PAO1 and ΔPf4 OMVs. Structures were predicted and calculated free energy obtaining using Vienna RNA fold. Representative structures from RNAfold output were overlayed. For **(C–E)** the number of replicates per condition was n = 3. All data are shown as the mean ± SD of at least three biological replicates with n ≥ 3 technical replicates per condition and experiment. *p < 0.05; a **p < 0.01; and ****p < 0.0001. Significance was assessed by ANOVA followed by Holm Šídák test for multiple comparisons.

To determine if *P. aeruginosa* OMVs contain small RNAs with the potential to activate TLR3 and type I IFN response, we extracted total RNA from OMV preparations. Prior to RNA extraction, OMV preparations were digested with proteinase K to remove proteins, followed by digestions with DNaseI to remove DNA which might co-precipitate with RNA (**Figure 3A**). The resulting purified RNA was analyzed by Bioanalyzer to quantify RNA size distributions (**Figure 3B**). In general, a relatively large proportion of OMV-packaged RNAs were small and at a length between 25 and 200 nucleotides (**Figure 3B**).

Next, we investigated whether purified OMV RNA was capable of inducing type I IFN responses. A human IFNβ ELISA was used to quantify titers in supernatants from human U937 macrophages treated with 100 ng/ml OMV RNA (**Figure 3C**). OMV RNA was digested overnight by benzonase, a non-specific endonuclease, as an additional control for specificity of IFN responses to RNA content. OMV RNA samples tested induced IFNβ to the same extent as the positive control poly (I:C), a TLR3 agonist. In contrast to that, benzonase-treated RNA induced a much-reduced IFNβ response, suggesting that the majority of the IFN generated was in response to OMV RNA (**Figure 3C**).

We sought to determine if OMV RNA packaged into lipid micelles mimicking OMV transport could induce a type I IFN response. Purified RNA was transfected into U937 macrophages using RNAfectamine, a cationic lipid transfection reagent which forms single lipid layer micelles around nucleic acids. Cells were incubated for 24 h, and supernatants assessed for type I IFN content using the HEK-IFNα/β reporter cell line. Mammalian mRNA and benzonase-digested OMV RNA were used as controls, and neither induced interferon to the extent of the intact OMV RNA (**Figure 3D**). As an additional control, we quantified residual endotoxin in OMV RNA preparations, amounting to an average of 15–20 ng/ml, and treated macrophages with an equivalent amount of LPS. The IFN signal induced by this endotoxin control was significantly lower than that of OMV RNA (data not shown), indicating that IFN responses are not driven by endotoxin contamination.

Finally, we assessed the ability of OMVs with or without Pf4 phage to stimulate TLR3 signaling, a receptor for dsRNA, using the HEK-TLR3 reporter cell line. We found that while intact OMVs alone induced some TLR3 activation, OMV and Pf4 together induced the strongest signal (**Figure 3E**). 2× PEG Pf4 alone and OMV in combination with mock preparation did not activate TLR3 to the same extent.

Thus, these data demonstrate that OMV-derived RNA trigger TLR3 resulting in type I interferon production.

### 3.4 Highly abundant small RNAs derived from OMVs are predicted to form stable hairpin structures

OMV RNA purified as described above (**Figure 3A**) was further fractionated by size to enrich for small RNAs (less than 200 nucleotides) and sequenced. Overrepresented sequences, each comprising more than 0.1% of the reads, were identified. Secondary structures of these sequences were predicted using the Vienna RNAfold tool (Lorenz et al., 2011), and predicted free energy of these structures was obtained. Structures were successfully predicted for all overrepresented sequences over a range of free energies with no significant difference in free energy distribution of PAO1 and PAO1ΔPf4 OMV RNAs (**Figure 3F**). Although these structures possessed relatively high free energy compared with highly stable RNA structures, such as tRNAs, there was a considerable overlap with the free energy distribution of all known miRNA structures (Thakur et al., 2011). These findings indicate that OMVs carry abundant quantities of small RNAs with stable secondary hairpin structure and the potential to engage TLR3.

### 3.5 Pf4/OMV complexes dampen macrophage activation

**Figure 4:**
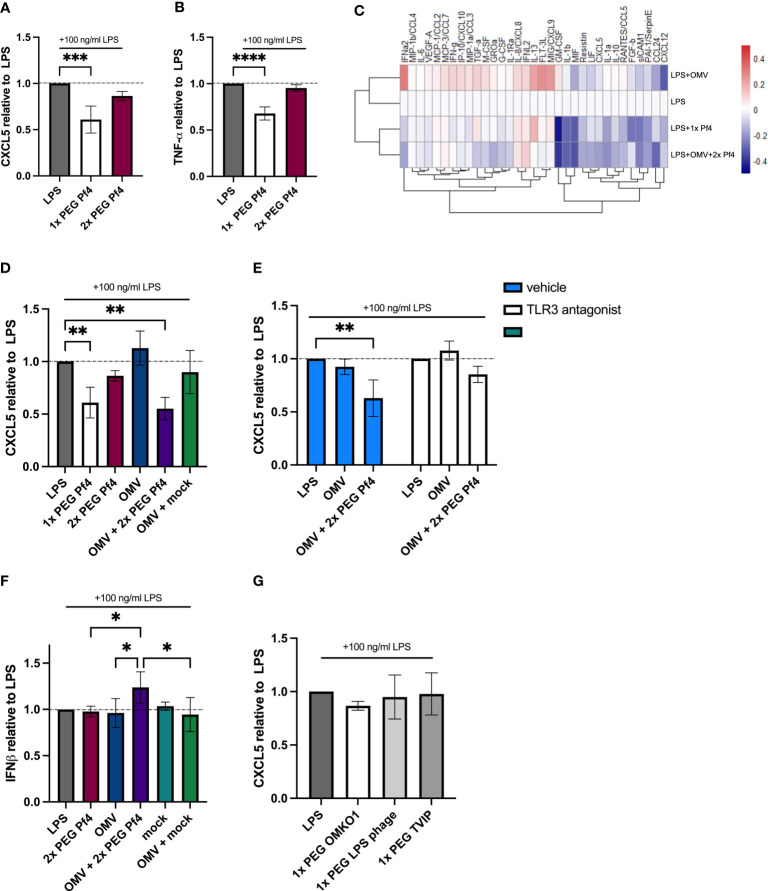
Pf4/OMV complex triggers a downregulation of inflammatory cytokine production and induction of type I IFN secretion. A and B Human U937 macrophages were stimulated with 100 ng/ml LPS and treated with 1× PEG or 2× PEG Pf4. **(A)** CXCL5 and **(B)** TNF-α levels of the supernatants were determined by ELISA. **(C)** Luminex analysis of supernatants from human U937 macrophages supplemented with 100 ng/ml LPS; OMVs; 10^9^ pfu/ml 1× PEG Pf4; or 10^9^ pfu/ml 2× PEG Pf4 + OMVs for 24 h. **(D)** Human U937 macrophages were treated the same way as in C for 24 h, and the supernatants were assayed for CXCL5 protein secretion by ELISA. **(E)** Human U937 macrophages were stimulated with 100 ng/ml LPS. PBS (vehicle control), or 27 µM of TLR3/dsRNA complex inhibitor along with Pf4, and/or OMVs were added to the cells and incubated for 24 h. CXCL5 levels in the supernatants were quantified by ELISA. **(F)** HEK-IFNα/β cells were stimulated with conditioned media from human U937 macrophages which had been stimulated with 100 ng/ml LPS and treated with 10 ng/ml OMVs, 10^9^ pfu/ml 2× PEG Pf4, or mock preparation for 24 h. IFN signal was determined by absorbance and normalized to the PBS control. **(G)** Human U937 macrophages were stimulated with 100 ng/ml LPS and treated with either 10^9^ pfu/ml 1× PEG OMKO1, LPS phage, or TIVP. CXCL5 levels were quantified by ELISA and normalized to the LPS control condition. The mean ± SD is shown with three biological replicates and n ≥ 2 replicates per condition. *p < 0.05; **p < 0.01; and ***p < 0.001; ****p < 0.0001. Significance was assessed by ANOVA followed by Holm–Šídák test for multiple comparisons.

Considering these results, we further hypothesized that the Pf4/OMV complex-dependent stimulation of TLR3 and type I IFN production would likely impact the production of pro-inflammatory cytokines. We further characterized the differing effects of 1× PEG and 2× PEG Pf4 preparations on macrophage chemokine productions and functions. We were intrigued to observe that the additional round of PEG treatment and removal of RNA by endonuclease digestion strongly reduced the ability of Pf4 phage to inhibit CXCL5 and TNF-α production in response to LPS (**Figures 4A, B**).

Moreover, we also investigated whether 2× PEG Pf4 decorated with OMVs (10 ng/ml LPS content) mimic the effects of 1× PEG Pf4 preparations. To this end, a Luminex assay was performed on supernatants derived from human U937 macrophages incubated for 24 h with various combinations of LPS, 1× PEG Pf4, OMVs, and 2× PEG Pf4 with OMVs (**Figure 4C**). All conditions were normalized to the LPS control. We observed that OMVs had an additive effect on inflammatory cytokines produced in response to LPS with increased production of IL-8, IFNγ, and other factors. This is consistent with pro-inflammatory effects previously attributed to OMVs in other settings (Perez Vidakovics et al., 2010; Park et al., 2013; Lee, 2019). Conversely, cells treated with LPS together with either 2× PEG Pf4 + OMVs or 1× PEG Pf4 had similar reductions in the levels of multiple cytokines compared with the LPS control (**Figure 4C**). We confirmed these findings vis-à-vis CXCL5 using additional control of a mock phage preparation. This mock phage preparation did not induce CXCL5 downregulation (**Figure 4D**).

We then assessed the effects of OMV-supplemented Pf4 on CXCL5 production and the dependency of this present system on TLR3 by treating human U937 macrophages with a small-molecule compound that specifically prevents dsRNAs from binding to TLR3 (Cheng et al., 2011) and found that CXCL5 downregulation in response to Pf4 was abolished in our LPS stimulation assay (**Figure 4E**).

To establish that Pf4/OMV complexes induce type I IFN production, we employed a HEF-IFNα/β cell line which reports on engagement of the IFNAR1/2 receptor. These HEK-IFNα/β cells were incubated with conditioned media from human U937 macrophages stimulated with various reagents for 24 h, and the degree of IFNAR stimulation was considered to be a proxy for IFNα/β concentration in the conditioned media. While 2× PEG Pf4, mock preparation, and OMVs did not induce appreciable type I IFN production above the LPS control, cells co-stimulated with LPS together with 2× PEG Pf4/OMV produced the biggest IFN response (**Figure 4F**). Cells incubated with a combination of LPS together with OMVs and mock preparation did not show an increase in type I IFN to the same magnitude (**Figure 4F**).

Finally, we also tested the impact of 1× PEG lytic phages that can infect *P. aeruginosa* for CXCL5 downregulation. We treated human U937 macrophages with LPS and phages for 24 h. Neither of the tested lytic phages were able to significantly reduce the CXCL5 levels compared with the LPS control condition (**Figure 4G**).

Taken together, these data indicate that Pf4/OMV complexes downregulate multiple pro-inflammatory cytokines in human macrophages, including neutrophil chemoattractants, in a TLR3- and IFNAR-dependent manner. Moreover, the pathway that results in CXCL5 inhibition is parallel to but not dependent on the effects of Pf4 we previously reported on TNFα (Sweere et al., 2019).

### 3.6 Pf4 impairs neutrophil recruitment by human macrophages

**Figure 5:**
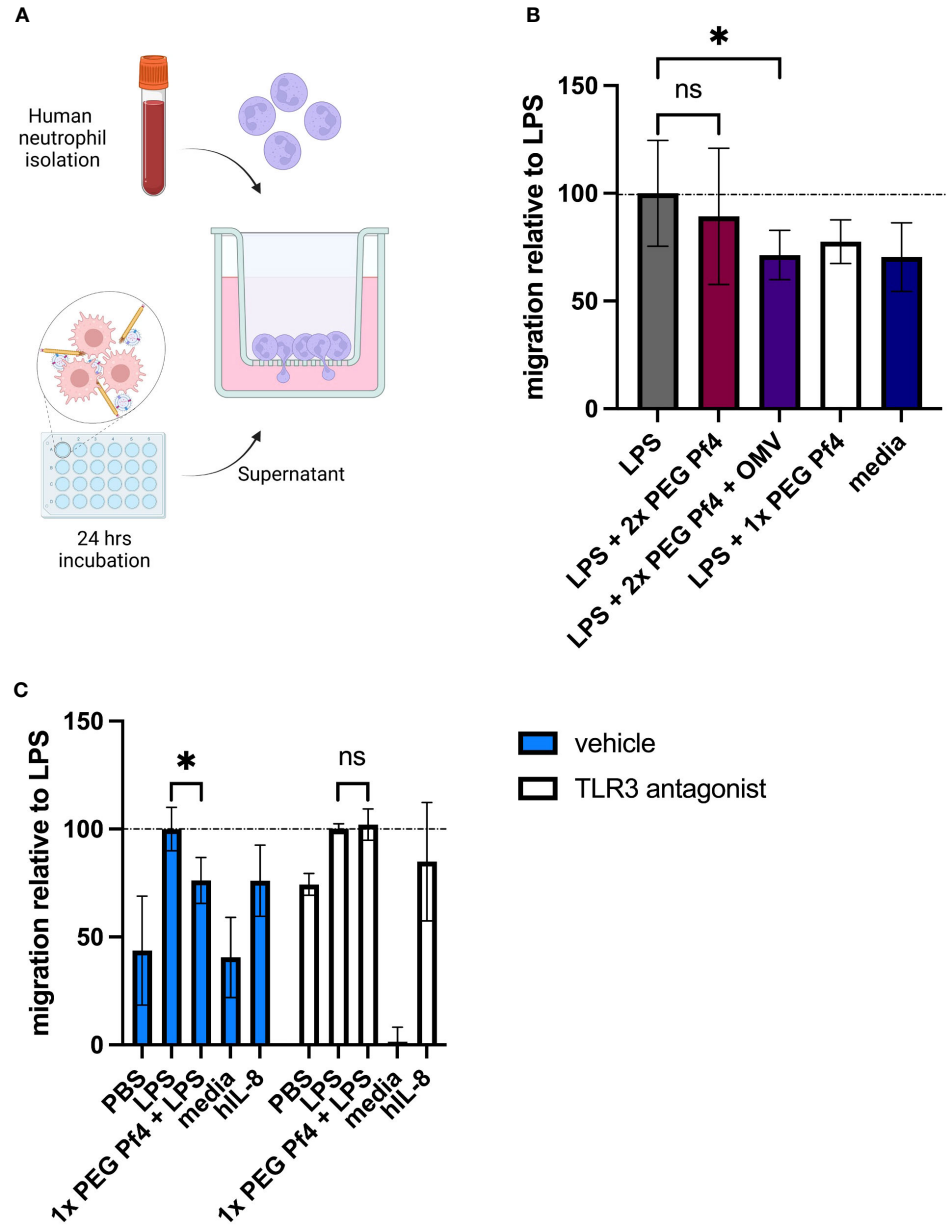
Pf4/OMV complexes impair neutrophil migration *in vitro*. **(A)** Schematic representation of the neutrophil migration assay. Primary human neutrophils were seeded onto Transwell inserts exposed to conditioned media derived from human U937 macrophages in the bottom well as chemoattractants. Neutrophil migration was tracked over 60 min and normalized to the LPS condition. **(B)** Primary human neutrophils were exposed to conditioned media from human U937 macrophages treated with PBS; LPS; LPS + 10^9^ 2× PEG Pf4 + OMV; LPS + 1× PEG Pf4; or media as a control. **(C)** Human U937 macrophages were treated with vehicle (PBS), or 100 µM TLR3/dsRNA complex inhibitor along with LPS; LPS + 1× PEG Pf4; media; or human IL-8 as a positive control. Primary human neutrophils were exposed to the supernatants from these U937 cells, and their migration was assessed after 60 min and normalized to LPS. All data were normalized to the internal LPS control condition unless otherwise specified. The mean ± SD is shown with three biological replicates and n ≥ 2 replicates per condition. *p < 0.05; ns, not significant. Significance was assessed by ANOVA followed by Holm–Šídák test for multiple comparisons, or by ANOVA followed by *t*-test with Bonferroni *post-hoc* test.

To test the functional consequences of reduced chemokine production, we adapted a neutrophil migration assay (Frevert et al., 1998) using freshly isolated human primary neutrophils from multiple donors and conditioned media from human U937 macrophages. Macrophages were exposed to differently purified Pf4 preparations along with LPS for 24 h to induce inflammatory cytokine production, and conditioned media from these cells was used as a chemoattractant in a Transwell setup (**Figure 5A**). Neutrophils were then stained with the live-cell dye calcein AM and tracked as they migrated through the 3-µm pores of the Transwell insert.

We found that conditioned media from cells treated with a combination of LPS and Pf4/OMV complexes induced less migration than cells treated with LPS alone (**Figure 5B**), indicating that the presence of Pf4 associated with OMV is sufficient to alter macrophage function in response to bacterial stimulation. Inhibition of TLR3 in macrophages resulted in loss of Pf4/OMV-driven reduced neutrophil chemoattraction as compared with the LPS control (**Figure 5C**). Finally, we used a small-molecule inhibitor CXCR2, the neutrophil-expressed receptor for CXCL5, as well as recombinant human CXCL5 to assess the dependency of this migration phenotype on these specific chemokines. We found that treating neutrophils with the CXCR2 inhibitor significantly reduced their chemoattraction to conditioned media across conditions, indicating that signaling through this receptor is required for neutrophil migration in our assay (data not shown). We further found that supplementation of 1× PEG Pf4 and LPS conditioned media with CXCL5 rescued migration to LPS control levels (data not shown).

Together, these data indicated that Pf4 reduced the ability of LPS to stimulate macrophages to induce granulocyte migration.

### 3.7 Pf4/OMV complexes and Pf4-overproducing *P. aeruginosa* elicit diminished neutrophil migration

**Figure 6:**
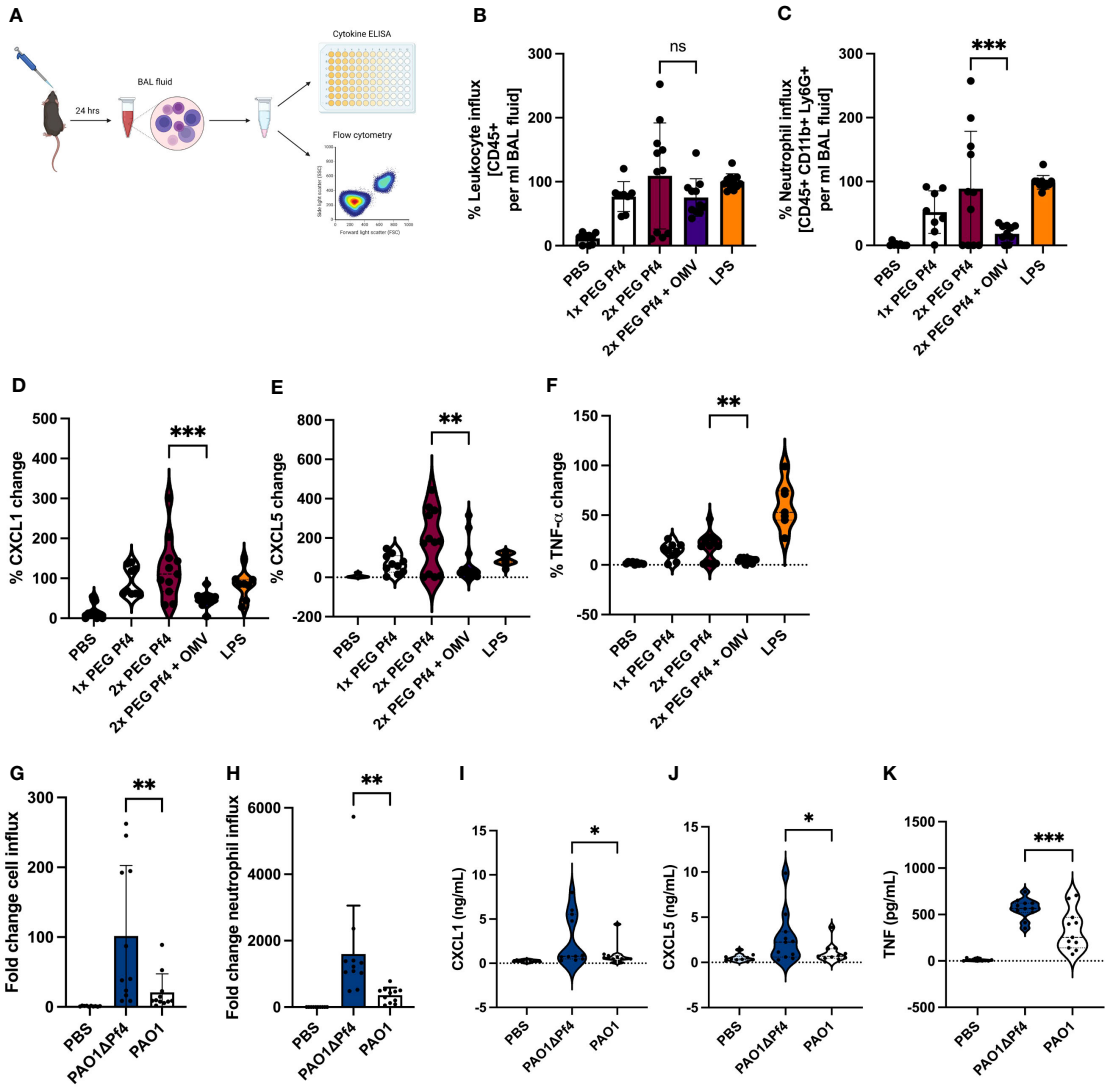
Neutrophil migration is reduced by Pf4/OMV complexes and Pf4 phage production in an acute *P. aeruginosa* pneumonia murine model. **(A)** Mice were inoculated intranasally for 24 h with either 10^9^ pfu/ml 1x PEG Pf4, 2x PEG Pf4, 2x PEG + OMVs, LPS, PBS, or live bacteria like PAO1 or ΔPf4. After 24 h, the animals were scarified and bronchoalveolar lavage (BAL) fluid was collected for cytokine measurments and cellular assessments via flow cytometry **(B–C, G–H)**. BAL fluid samples were evaluated for **(B, G)** total leukocyte cell content (CD45^+^ cells), and **(C, H)** for neutrophil quantities (CD45^+^, CD11b^+^, Ly6G^+^ cells). **(D–F, I–K)** BAL chemokine quantifications **(D, I)** for CXCL1, **(E, J)** for CXCL5, and **(F, K)** for TNF-α. Data are displayed as the mean ± SD from three individual experiments with n ≥ 9 mice per experiment with live bacteria. *p < 0.05; **p < 0.01; and ***p < 0.001. Significance was assessed by ANOVA followed by Holm–Šídák test for multiple comparisons. Of note, the comparisons to PBS (the negative control condition) are significant for all of the conditions involving either LPS treatment or bacterial infection. These p-values were not shown because the large number of comparisons this involves would distract from the more meaningful comparisons.

To obtain a more complete picture of Pf4/OMV complex-induced cytokine changes to bacterial stimulation, we intranasally inoculated mice with 1× PEG Pf4, 2× PEG Pf4, 2× PEG Pf4 + OMV, or LPS. After 24 h, we sacrificed the animals and collected bronchoalveolar lavage (BAL) fluid and quantified the cell influx into the lung with flow cytometry, as well as the chemokine levels with ELISA (**Figure 6A**). Mice inoculated with 2× PEG Pf4 showed a significantly higher neutrophil influx and pro-inflammatory cytokine production in the lung compared to mice treated with 2× PEG Pf4 + OMV (**Figures 6B–F**). The treatment of mice with Pf4/OMV complexes resulted in decreased levels of CXCL1, CXCL5, and TNF-α compared with mice treated with Pf4 lacking OMVs (**Figures 6D–F**).

Building on our previous finding that treatment with *P. aeruginosa* strain PAO1 supplemented with Pf phage reduced pulmonary inflammation in response to bacterial infection (Secor et al., 2017), we examined *P. aeruginosa* infection in the absence of Pf4 phage. To this end, we treated mice with PAO1 or an isogenic strain lacking Pf4, PAO1ΔPf4. These strains have comparable growth curves (Sweere et al., 2019) such that differences between them cannot be attributed to altered growth. After 24 h, BAL fluid was collected and evaluated for cell influx and chemokine/cytokine content (**Figure 6A**). Mice infected with PAO1ΔPf4 showed significantly greater neutrophil influx and pro-inflammatory cytokine production than mice infected with Pf4-producing PAO1 (**Figures 6G–K**). PAO1-infected mice also exhibited significantly lower levels of CXCL1, TNF-α, and CXCL5 in BAL as compared with PAO1ΔPf4-infected mice (**Figures 6I–K**).

Together, these data demonstrate that Pf4/OMV complexes are associated with diminished neutrophil influx in this model in conjunction with reduced production of pro-inflammatory cytokines.

### 3.8 A model for how Pf4 phage and OMVs alter the innate immune response to bacterial stimulus LPS

Our findings presented in this study establish that Pf4 phages complex with OMVs and inhibit macrophage activation in response to bacterial stimuli like LPS, resulting in impaired production of neutrophil chemotactic factors and less neutrophil migration during *P. aeruginosa* infections (**Graphical abstract**). Our data suggest that as Pf4 is extruded from its bacterial host, it associates with RNA-loaded OMVs. These Pf4/OMV complexes are endocytosed by lung-resident myeloid cells such as macrophages. RNA with hairpin secondary structure bound to the surface of OMVs triggers TLR3 activation. This induces type I IFN production, which inhibits pro-inflammatory cytokine production in response to other bacterial products. Among the cytokines inhibited are neutrophil chemoattractants such as CXCL5 which results in abrogated neutrophil influx to the site of *P. aeruginosa* infection and impaired bacterial clearance.

## 4 Discussion

We report that Pf4 phage inhibits macrophage responses to LPS and that this effect is dependent on bacterial debris bound to phages. In particular, we demonstrate that macrophages cultured with 1× PEG Pf4 phage preparations downregulate multiple LPS-induced factors, including the potent neutrophil chemoattractant CXCL5. These suppressed macrophages are also less effective at inducing neutrophil migration in a mouse model of acute *P. aeruginosa* lung infection. Neutrophil influx to sites of *P. aeruginosa* infection is critical to control and clear an infection (Koh et al., 2009; Lavoie et al., 2011). The findings we present in this work indicate that the presence of Pf4 phage and OMVs could be associated with negative outcomes in patients infected with *P. aeruginosa*, through ineffective macrophage activation and subsequent impaired recruitment of neutrophils at early stages of infection.

We find that OMVs bound to Pf4 phage can trigger innate immune recognition in ways that are distinct from responses to either Pf4 phages or OMVs alone. In particular, we find that Pf4-associated OMVs carry small RNAs that are capable of triggering TLR3 responses and IFNβ production by human cells. It is well-described that type I IFN exert an anti-inflammatory effect on immune cells, inhibiting the production of cytokines and chemokines necessary for effective antibacterial responses (Tian et al., 2012; Lee et al., 2015; Connolly and Hussell, 2020). Prior studies have demonstrated that the RNA composition of OMVs appears to selectively include small RNAs (<60 nucleotides) with potential selection and enrichment of certain RNAs to suggest active export and packaging (Ghosal et al., 2015; Sjöström et al., 2015; Augustyniak et al., 2022). We confirm here that OMVs are enriched in small RNAs predicted to form stable hairpin structures and that these RNAs are likely to be responsible for the TLR3 dependency of the Pf4/OMV complex effect on macrophages.

These data suggest that our previous report showed that Pf4 phage triggers TLR3 and type 1 interferon-mediated inhibition of TNF-α may also have been mediated by OMV bound to phage (Sweere et al., 2019). In that study, we detected phage RNA present in Pf4 phage preparations that had been taken up by human cells. With the benefit of hindsight, it seems likely that RNA bound to and contained within OMV was responsible. The protocol modification of substituting benzonase (which degrades RNA) for DNase (which does not) and the consistent use of two rounds of PEG treatment during our phage preparations allows us to generate “clean” preparations in this study.

Although we chose to focus on the interaction between macrophages and neutrophils due to the observed downregulation of several neutrophil chemoattractants, several other cytokines altered by Pf4-OMV complex stimulation may affect the course of an immune response to *P. aeruginosa*. In particular, the IL-1α/β and GM-CSF axis has been shown to be important for neutrophil longevity and thereby effective bacterial clearance (Bober et al., 1995; Fossati et al., 1998; Laan et al., 2003). In addition, GM-CSF has been shown to amplify bystander phagocyte cytokine production in *Legionella* infection (Liu et al., 2020).

The coexistence of filamentous phages with the bacterial hosts they infect suggests a potential symbiotic relationship. Indeed, prior work has demonstrated that Pf4 impacts *P. aeruginosa* pathogenesis during chronic pulmonary and wound infections (Secor et al., 2015; Secor et al., 2017; Burgener et al., 2019; Sweere et al., 2019). Unlike lytic phages which lyse the bacterial host cell, chronically infecting phages like Pf4 integrate their genetic material into the bacterial genome and coexist with the bacteria in high titers. This coexistence assures that continued interaction between the phage and the bacterial cell will continue, manifesting in such phenomena as liquid crystal formation using bacterial polymers and phage-formed occlusive sheaths that protect bacterial cells from antibiotics (Secor et al., 2015; Tarafder et al., 2020). We present here a novel instance of Pf4-*P. aeruginosa* symbiosis: association of the Pf4 particle with *P. aeruginosa* OMVs produced simultaneously by the bacterium. The immunologic responses described here are dependent on the physical association of OMVs with Pf4. It is well known that phages co-purify with molecules and macromolecular structures from the bacterial cell wall and outer membrane, including endotoxins and other immunogenic bacterial products. Indeed, strategies of phage purification to obtain phages free of bacterial debris are a priority for facilitating phage therapy treatment protocols. Interestingly, OMVs were recently shown to adhere to *P. aeruginosa* lytic phages as a defense against phage predation, but the immune consequences of this were unknown (Augustyniak et al., 2022). We show here that Pf4/OMV association is critical for reducing macrophage activation in response to bacterial components.

Our work leaves open several questions related to the types of interactions that drive OMV association with phages. Given that Pf4 phages have an overall strong negative charge, and OMVs are also negatively charged as a consequence of their LPS content, charge–charge interactions are unlikely to facilitate this interaction. The tendency of OMVs to localize at the end of the phage particle suggest a possible interaction with Pf4 tail proteins. This could be a bacterial protective strategy, given that Pf phages infect bacterial hosts using tail-localized proteins (Mai-Prochnow et al., 2015).

We find that OMVs bound to Pf4 phage can trigger innate immune recognition in ways that are distinct from responses to either Pf4 phages or OMVs alone. In particular, we find that Pf4-associated OMVs carry small RNAs that are capable of triggering TLR3 responses and IFNβ production by human cells. It is well described that type I IFN exert an anti-inflammatory effect on immune cells, inhibiting the production of cytokines and chemokines necessary for effective antibacterial responses (Tian et al., 2012; Lee et al., 2015; Connolly and Hussell, 2020). Prior studies have demonstrated that the RNA composition of OMVs appears to selectively include small RNAs (<60 nucleotides) with potential selection and enrichment of certain RNAs to suggest active export and packaging (Ghosal et al., 2015; Sjöström et al., 2015). We confirm here that OMVs are enriched in small RNAs predicted to form stable hairpin structures, and that these RNAs are likely to be responsible for the TLR3 dependency of the Pf4-OMV complex effect on macrophages.

Future avenues of investigation include determining whether Pf4 phages isolated from sputum form individuals with cystic fibrosis contain OMVs and whether a correlation exists between patients with the highest OMV sputum content and worst infection control outcome.

Finally, our findings prompt the question of whether other impure phage preparations likewise regulate mammalian immunity. The possibility that other phages likewise affect immune responses to commensal and pathogenic bacteria alike is an intriguing possibility that merits continued research.

## Data availability statement

The datasets presented in this study can be found in online repositories. The names of the repository/repositories and accession number(s) can be found below: NCBI, PRJNA994600.

## Ethics statement

Ethical approval was not required for the studies on humans in accordance with the local legislation and institutional requirements because only commercially available established cell lines were used. The animal study was approved by Stanford University Animal Welfare Board/IACUC. The study was conducted in accordance with the local legislation and institutional requirements.

## Author contributions

NP, MP, and PB contributed to conception, design, and supervision of the study. NP, MP, PB, GK, and AE were responsible for methodology. NP, MP, FD, NH, AH, PJ, AE, and GK were involved in the investigation and experimental performance. NP and MP wrote all sections of the manuscript. All authors contributed to the article and approved the submitted version.
